# Resilient botany: Innovation in the face of limited mobility and resources

**DOI:** 10.1002/aps3.11577

**Published:** 2024-04-02

**Authors:** Gillian H. Dean, N. Ivalú Cacho, Alejandro Zuluaga Trochez, Gregory J. Pec

**Affiliations:** ^1^ Independent researcher; ^2^ Instituto de Biología Universidad Autónoma de México México City Mexico; ^3^ Departamento de Biología Universidad del Valle Cali Colombia; ^4^ Department of Biology University of Nebraska at Kearney Kearney 68849 Nebraska USA

In 2020, at the beginning of the COVID‐19 pandemic, *Applications in Plant Sciences* (*APPS*) published a special issue titled “Conducting botanical research with limited resources: Low‐cost methods in the plant sciences” (Dean et al., 2020). The goal of that collection was to highlight robust, low‐cost methods that could be used by researchers in under‐resourced settings. Plant scientists face resource limitations for many reasons. In some countries, inadequate national funding is a major issue, as well as limited long‐term investment in research infrastructure. At the same time, factors contributing to low‐resource settings are also experienced by researchers in countries where research funding is more abundant. For example, although national research programs in the Global North are generally well funded, these funds may be difficult to access for investigators who hold positions at smaller institutions, such as predominantly undergraduate institutions.

Regardless of geographic location, lack of training and access to expensive and specialized equipment can be limiting, and funding to acquire expensive equipment often does not include salary for maintenance and operation by trained personnel after the initial purchase and set up. In addition, research may be performed by new investigators such as undergraduate or graduate students, or when established labs wish to explore new areas of research or techniques without committing significant resources.

Globally, substantial and significant scientific research is performed in these under‐resourced settings. The COVID‐19 pandemic exacerbated this situation by disrupting supply chains and forcing researchers to work from home or in isolation at their workplace, resulting in dynamic adaptations in their approaches to generating or collecting data. Suddenly confronted with an inability to travel, many scientists looked to their local environment to leverage their skills to continue research closer to home. In light of these challenges, this *APPS* special issue, titled “Resilient botany: Innovation in the face of limited mobility and resources,” showcases the creative ways that plant scientists carried on with research during a global pandemic. The papers in this issue encompass a variety of fields and scales of research, ranging from investigations of plant structure at the microscopic level to utilizing big data to understand biodiversity, but they all have one thing in common: they are all accessible to researchers and practitioners challenged by funding or travel restrictions.

It has been well documented that cataloging global biodiversity is a daunting task that will take a concerted effort by many scientists and community scientists, especially given that much of the world's biodiversity is located in areas with under‐resourced research communities. A large amount of data is already contained in herbaria, and digitizing this information is an important milestone toward improving access. The R package gatoRs presented by Patten et al. (2024) in this special issue addresses challenges in obtaining and processing digitized biodiversity data by offering a streamlined workflow. The package includes functions for downloading records from the Global Biodiversity Information Facility (GBIF) and Integrated Digitized Biocollections (iDigBio) and a cleaning function for specimen records. Notably, gatoRs accommodates variations in download structures between GBIF and iDigBio and allows user control through interactive cleaning steps. The pipeline aims to make biodiversity data processing efficient and accessible and is expected to benefit both experienced and novice users in the scientific community. Additionally, the package is a valuable tool for introducing classroom biodiversity concepts through herbarium specimens.

An often‐overlooked component of biodiversity is the microbiota, the vast array of microorganisms that inhabit every corner of our world. These microscopic organisms play crucial roles in various ecosystems, contributing to nutrient cycling, decomposition of organic matter, and plant establishment and succession (Smith and Read, 2008; Diamini et al., 2022). Despite their importance, our understanding of microbiota remains limited, largely due to the challenges associated with studying them. In this context, the second paper in this issue introduces a new, cost‐effective moist chamber culture technique designed to recover a broader spectrum of microbial species (Bordelon et al., 2024). This technique emphasizes its low‐cost, accessible approach, addressing the need for more cost‐effective research methods and the involvement of community scientists. Bordelon et al. (2024) provide a robust discussion on the environmental factors affecting microbiota on live tree bark, including examples of microbiota found on rough bark of live trees and considerations for smooth bark live trees in the context of myxomycete cultures. The importance of pH, water‐holding capacity, and bark characteristics is explored, providing insights into the diversity and abundance of myxomycetes associated with different tree species (Bordelon et al., 2024).

In modern agriculture, the integration of advanced technologies such as remote sensing (Yang, 2020), DNA‐based detection methods (Martinelli et al., 2015), and robotics and artificial intelligence (Balaska et al., 2023) has become increasingly important for enhancing crop management and mitigating the impact of plant diseases (Spadaro and Gullino, 2019). One prominent area of innovation is the application of computer vision (CV) (Ouhami et al., 2021). This dynamic field leverages the capabilities of machine learning to revolutionize the way we detect and respond to diseases affecting crops (Ahmed and Reddy, 2021). The significance of addressing plant pathogens is underscored by their potential to cause catastrophic consequences such as famine and economic collapse by rendering local cultivation unprofitable (Dai and Fan, 2022). The third paper in this issue provides a focused review on the increasing use of CV for precise disease detection and optimal pesticide application timing, featuring case studies from agricultural practices in cocoa production. Here, Sykes et al. (2024) underscore the advantages of CV, including cost reduction and prevention of misapplication, discussing financial and ecological implications. The analysis goes beyond previous work, exploring technical concepts in applying CV to plant pathology, training data acquisition, and insights into machine learning methods. The authors highlight the significance of curated training data, exploring computationally efficient techniques and providing a comparative analysis of model architectures. Furthermore, Sykes et al. (2024) discuss the evolving role of machine learning in optimizing pesticide application and provide a comprehensive perspective on applying CV in plant pathology, including practical considerations for data gathering and a roadmap for commercial implementation.

In addition to these large‐scale methods, there is also scope for developing reliable, inexpensive, and easily accessible protocols for performing laboratory‐based experiments. In this special issue, we highlight two simple and robust plant anatomy and physiology methods for settings with fewer resources and limited infrastructure.

Koch et al. (2024) expand on a method first described by Díaz Dominguez et al. (2022) for measuring lichen thalli hydrophobicity that requires only a micropipette, distilled water, a tripod, and a phone or camera, thereby providing a cost‐effective and rapid way of assessing this functional trait. By analyzing 93 lichen taxa, the authors’ results support the method's efficacy in capturing a spectrum of hydrophobicity levels, including both highly hydrophilic and highly hydrophobic species. This method, which can be used with both fresh and archival material, emerges as a valuable and versatile tool for ecophysiology‐based lichen trait assessment across climates, lichen species, and growth forms, and offers insights into its potential as a predictor of climate change effects.

The sectioning of plant material is a classic method that has been used extensively to obtain details of a wide variety of tissue structures, with applications in taxonomy and plant anatomy (for a comprehensive overview, see Yeung et al., 2016). Angeles and Madero‐Vega (2024) share a low‐cost sectioning medium created by dissolving biaxially oriented polypropylene (BOPP) in a commercially available polyurethane remover. The resulting BOPP syrup can be used for plant tissue sectioning, as well as for obtaining surface prints of both wood and leaf parts. BOPP is a plastic material widely used in the food industry for wrapping produce and other food items, and is usually seen as a waste product. Therefore, it is an attractive starting material for this low‐cost method as it is widely available at no cost in many countries.

In summary, this special issue presents affordable and innovative approaches addressing challenges in biodiversity cataloging, microbiota discovery, agriculture, anatomy, and physiology. The gatoRs R package streamlines access to and use of digitized biodiversity data, while a cost‐effective moist chamber culture technique enhances our understanding of microbiota. The integration of computer vision in agriculture, discussed by Sykes et al. (2024), demonstrates its potential for precise disease detection and pesticide application. Furthermore, the issue showcases accessible methods for lichen thalli hydrophobicity assessment and plant tissue sectioning, emphasizing simple and cost‐effective ways to advance research in plant physiology and anatomy. Together, these contributions offer valuable insights and practical tools for researchers, educators, and community scientists in resource‐limited settings. [Correction added on 8 April 2024 after first online publication: In the last paragraph, the sentence “The gatoRs R package streamlines the digitization of biodiversity data,…” was inaccurate. The sentence has been corrected.]

## AUTHOR CONTRIBUTIONS

G.J.P. and G.H.D. initiated this special issue, and N.I.C. and A.Z.T. contributed to its development. G.J.P., N.I.C., and A.Z.T. acted as handling editors for manuscripts. G.H.D. wrote the first draft of this article, with contributions from all other authors.
